# Pharmacodynamics, Pharmacokinetics, and Antiviral Activity of BAY 81-8781, a Novel NF-κB Inhibiting Anti-influenza Drug

**DOI:** 10.3389/fmicb.2017.02130

**Published:** 2017-11-02

**Authors:** Karoline Droebner, Emanuel Haasbach, Sabine E. Dudek, Gerhard Scheuch, Karlheinz Nocker, Sebastian Canisius, Christina Ehrhardt, Georges von Degenfeld, Stephan Ludwig, Oliver Planz

**Affiliations:** ^1^Interfaculty Institute for Cell Biology, Department of Immunology, Eberhard Karls University, Tübingen, Germany; ^2^Friedrich Loeffler Institut, Tübingen, Germany; ^3^Bayer Pharma AG, Pharmaceuticals, Therapeutic Research Groups, Cardiovascular Research, Wuppertal, Germany; ^4^Institute of Virology Muenster, Westfälische Wilhelms-Universität Münster, Münster, Germany; ^5^GS Bio-Inhalation GmbH, Gemünden am Main, Germany; ^6^Ventaleon GmbH, Gemünden am Main, Germany

**Keywords:** influenza A virus, antivirals, NF-κB, aspirin, cellular targets, mouse model

## Abstract

Influenza is a respiratory disease that causes annual epidemics. Antiviral treatment options targeting the virus exist, but their efficiency is limited and influenza virus strains easily develop resistance. Thus, new treatment strategies are urgently needed. In the present study, we investigated the anti-influenza virus properties of D,L-lysine acetylsalicylate ⋅ glycine (BAY 81-8781; LASAG) that is approved as Aspirin i.v. for intravenous application. Instead of targeting the virus directly BAY 81-8781 inhibits the activation of the NF-κB pathway, which is required for efficient influenza virus propagation. Using highly pathogenic avian influenza virus strains we could demonstrate that BAY 81-8781 was able to control influenza virus infection *in vitro*. In the mouse infection model, inhalation of BAY 81-8781 resulted in reduced lung virus titers and protection of mice from lethal infection. Pharmacological studies demonstrated that the oral route of administration was not suitable to reach the sufficient concentrations of BAY 81-8781 for a successful antiviral effect in the lung. BAY 81-8781 treatment of mice infected with influenza virus started as late as 48 h after infection was still effective in protecting 50% of the animals from death. In summary, the data represent a successful proof of the novel innovative antiviral concept of targeting a host cell signaling pathway that is required for viral propagation instead of viral structures.

## Introduction

Influenza viruses are the causative agents of influenza, an acute respiratory disease of the upper and/or lower respiratory tract (Herold et al., 2015). Systemic signs such as fever, headache, myalgia, and weakness are the major symptoms of influenza. Annual epidemics, peaking in the winter, are regularly associated with excess morbidity and mortality, usually expressed in the form of excess rates of pneumonia and influenza-associated hospitalizations and deaths.

Influenza virus can be first detected within 24 h before the onset of disease, although the time between the incubation period and the onset of disease can vary from 18 to 72 h. The virus titer is rapidly rising and titers remain elevated for 24–48 h. Thereafter, titers rapidly decrease and after 5–10 days of viral shedding, the virus is no longer detectable.

Annual vaccinations using inactivated influenza A and B viruses derived from strains that circulated during the previous influenza season are the major public health measures for prevention of influenza. Regarding therapeutic intervention, there are two groups of currently licensed antiviral drugs for chemoprophylaxis and for the management of influenza with two different modes of action; either inhibition of M2 ion channel protein, or inhibition of the neuraminidase surface glycoprotein, respectively (Hayden, 2009). Amantadine and rimantadine inhibit the M2 membrane protein ion-channel activity of influenza A viruses, thus interfering with virus uncoating and inactivating of newly synthesized viral hemagglutinin. Amantadine and rimantadine have no effect on influenza B viruses. Neuraminidase inhibitors like zanamivir, oseltamivir, and peramivir are effective against both, influenza A virus (IAV) and influenza B virus (Govorkova et al., 2009; Gaur et al., 2010; Jackson et al., 2011; Baranovich et al., 2014). Zanamivir is administered through inhalation while oseltamivir is readily absorbed from the gastrointestinal tract and is therefore administered orally (Jackson et al., 2011; Yoshino et al., 2015). Peramivir was licensed in 2014 as a new neuraminidase inhibitor for intravenous medication (Yoshino et al., 2015).

The emergence of viral resistance is a common problem, especially when the use of antiviral drugs become widespread (Tosh et al., 2011). A large variety of influenza virus strains already are resistant against amantadine and its derivates. Resistance is also emerging against neuraminidase inhibitors (Yen et al., 2005; Tashiro et al., 2009; Spanakis et al., 2014). While the rates of neuraminidase inhibitors resistance are generally still low, a subtype wide resistance of seasonal H1N1 strain emerged in 2007–2009 in the Northern hemisphere (Burnham et al., 2013; Dapat et al., 2013), highlighting the continuous threat that neuraminidase inhibitors might share the same resistance fate as amantadines.

Thus, there is a high unmet medical need for an influenza treatment, which is less prone to induce viral resistance and is effective against all relevant sub-types and strains of influenza viruses, including newly emerging viruses and strains resistant to currently available antivirals.

Acetylsalicylic acid (ASA) has antiviral effects against influenza viruses *in vitro* and *in vivo* by inhibiting the intracellular transport and release of mature and infectious viral particles (Huang and Dietsch, 1988; Mazur et al., 2007). Due to the cellular mechanism of action, the development of resistant influenza strains is not expected (Nimmerjahn et al., 2004; Schmolke et al., 2009; Muhlbauer et al., 2015). It has been shown that this antiviral action is due to the inhibition of NF-κB by ASA, specifically inhibiting the NF-κB activating kinase IKKβ (Yin et al., 1998). Inhibition of NF-κB in virus infected cells leads to impaired expression of pro-apoptotic factors such as TRAIL and FasL and thereby prevents caspase-mediated nuclear export of viral ribonucleoproteins (vRNP), leading to their accumulation in the nucleus and failure to release mature, infectious viral particles (Wurzer et al., 2003, 2004; Muhlbauer et al., 2015). Thus, ASA indirectly targets an “Achilles heel” of replication in the cell without any direct action on an influenza viral target. While this may suggest that ASA might be an attractive novel drug candidate against influenza, the drug has two major disadvantages. First, it is known from published data, that ASA has limited pharmacokinetics, thus, oral administration of the compound wouldn’t lead to effective concentrations in the lung (Nagelschmitz et al., 2014). Second, there are also problems with an inhaled treatment since pure ASA would lead to respiratory irritation due to its acidic property and it is also reported to cause asthmatic symptoms (Hoak, 1983; Le Pham et al., 2017).

Stability and tolerability of inhaled ASA can be improved with a basic amino acid such as D,L-lysine, which prevents ASA from hydrolysis and converts it into a salt. Discoloration is prevented when ⋅ glycine is added. D,L-lysine acetylsalicylate ⋅ glycine (BAY 81-8781; LASAG) is licensed as Aspirin i.v. for intravenous medication. It is also known as *Aspirin inhale* since it can be safely administered as aerosol without the respiratory side effects described, except in asthmatic patients, where it can lead to intolerance (Kim et al., 2010). BAY 81-8781 dissociates readily into ASA and the two amino acids lysine and glycine upon dissolution in aqueous media. The amino acids lysine and glycine are both essential amino acids, which are part of the daily nutrition and necessary for physiological protein synthesis. They are considered to have no relevant pharmacodynamics- or toxic effects.

In the present work, we questioned whether BAY 81-8781 would have the same antiviral properties as ASA *in vitro* and whether treatment via the inhaled route would confer antiviral properties. Experiments were performed to test, whether BAY 81-8781 is effective against different virus strains of avian and human origin. Moreover, the antiviral activity was investigated in a mouse infection model in detail. From the experiments, we conclude that BAY 81-8781 is effective in cell culture and, when given via the inhaled route, is an effective antiviral compound also in the mouse model.

## Materials and Methods

### Viruses

Highly pathogenic H5N1 avian influenza A/Mallard/Bavaria/1/2006 (H5N1; MB1) virus; highly pathogenic avian influenza A/FPV/Bratislava/79 (H7N7; FPV) virus; human H1N1 pandemic strain A/Regensburg/D6/2009 (H1N1pdm09; RB1), human H5N1 strain A/Thailand/KAN-1/2004 were grown on Madin–Darby canine kidney cells (MDCK.2 ATCC: CRL-2936). The avian H5N1 subtype was originally obtained from the Bavarian Health and Food Safety Authority, Oberschleissheim, Germany. The avian IAV A/Bratislava/79 (FPV, H7N7) was originally provided by the strain collection at the Institute of Virology, Justus-Liebig University, Giessen, Germany. H1N1pdm09 was received from the Robert Koch-Institute, Berlin, Germany. All avian influenza A viruses and H1N1pdm09 were further propagated at the Friedrich-Loeffler-Institut, Friedrich Loeffler Institut for Animal Health, Tuebingen, Germany. H1N1pdm09 was also propagated at the University of Tuebingen; Interfaculty Institute of Cell-Biology. Experiments with H5N1 and H7N7 were performed under BSL3 conditions and H1N1pdm09 studies under BSL2 conditions.

### Ethics Approval and Mice

The animal studies were carried out in accordance with the recommendations of the German animal law (TierSchG). The protocol (FLI-340) was approved by the committee of the Regierungspraesidium Tuebingen, Germany. Female C57BL/6 mice at the age of 6–8 weeks (20–22 g) were obtained from the animal breeding facilities at the Friedrich Loeffler Institut, Federal Research Institute for Animal Health, Tuebingen, Germany.

### Antiviral Compounds

D,L-lysine acetylsalicylate ⋅ glycine (BAY 81-8781, LASAG; MW = 363.5), acetylsalicylate (ASA; MW = 180.1) and salicylate (SA; MW = 160.1) were provided by Bayer HealthCare AG (Wuppertal, Germany). For *in vitro* studies, directly prior to the experiment a 10 mM stock solution was prepared by dissolving 10.9 mg BAY 81-8781 in 3 mL BA-medium. For ASA, a 10 mM stock solution was prepared by dissolving 5.4 mg ASA in 1 mL PBS by 5 min incubation at 37°C. pH-value was adjusted to 7.4 with 1N NaOH (Riedel-de-Haën, Germany). Thereafter, 2 mL BA-medium were added to the ASA/PBS-solution. 4.8 mg SA was dissolved in 3 mL BA-medium to obtain a 10 mM stock solution. For *in vivo* studies, different amounts of BAY 81-8781 were dissolved immediately prior to experiment in always 35 mL ddH_2_O (0.3% = 0.105 g; 0.1% = 0.35 g; 3% = 1.05%; 10% = 3.50 g and 30% = 10.5 g). As a source for oseltamivir, the complete contents of a Tamiflu^®^ capsule was dissolved in 75 mL ddH_2_O to get a 1 mg/mL concentration.

### *In Vivo* Application

Mice received BAY 81-8781 via inhalation in a directed-flow nose-only exposure system provided by Bayer Health Care. This chamber is designed and operated so that BAY 81-8781 is dynamically delivered to each of the four exposure ports and exhaled air from exposed animals is immediately exhausted without the possibility of other animals rebreathing this atmosphere (Pauluhn and Thiel, 2007). Each treatment was started with the application of ddH_2_O to the control group followed by the ventilation of four mice with BAY 81-8781 solution. Prior to the daily applications, the chamber settings were controlled. The air flow was compressed to 3.6 bar and the chamber venting was achieved by a vacuum pump (Type: BS 2208, G & C Machines). The flow rate of the exhaust air (18 L/min) as well as the flow rate of the analysis extracting unit (1.1 L/min) was controlled with a flow meter (TSI 4000, PM no.: PM0114, TSI Incorporated). Prior to insertion, mice were labeled with numbers to ensure that they were always treated in the same position of one ventilation tube of the chamber. The supply of BAY 81-8781 to the chamber at a rate of 1.4 mL/min was performed by means of a peristaltic pump (Mini Plus 2, Gilson). Mice of each group were treated for roughly 25 min under the above-mentioned conditions until the volume of 35 mL was used up. The chamber and the nozzle were thoroughly cleaned after each run. To control and calculate deposition two analyses (5 and 10 min) of the air-flow were taken with a glass fiber filter (SM 13400-50, Sartorius), which was in the extraction unit. The glass fiber filter was weighed prior and after the analysis. After measurement glass fiber filters were removed and dried for 30 min at 70°C in a drying cabinet (Type: SUT 6420, Heraeus). The dried filters were capped in a silica gel-filled desiccator for about 10 min thereafter cooled and finally reweighted. For pharmacokinetic studies BAY 81-8781 was either applied via inhalation as described above, via intravenous injection into the tail vein or via the oral route. For determination of lung virus titer 24 h after infection and PK mice received a single treatment. For EC_50_ and survival experiment the animals were treated twice daily. Tamiflu^®^ was given twice daily (BID) *per os* via gavage in a concentration of 5 mg/kg bodyweight (BW). The application volume was 5 mL/kg BW.

### Pharmacokinetic Analyses

Acetylsalicylic acid is not stable in plasma due to the rapid hydrolysis by esterases present in plasma. To prevent further hydrolysis of ASA, 3 mg/mL sodium fluoride, a non-specific esterase inhibitor, was added to the blood samples at the time point of sampling. Samples were also cooled immediately. The determination of ASA was done in plasma after protein precipitation including a stable isotope labeled internal standard and separation by liquid chromatography and tandem mass spectrometric detection (LC-MS/MS). Determination of SA was done in plasma after protein precipitation including 4-acetylbenzoic acid as internal standard and separation by liquid chromatography and UV detection (HPLC-UV). The sample preparation of the lung tissue samples was done by homogenization of one part of exsanguinated lung tissue with five parts 0.9% NaCl also including 3 mg/mL sodium fluoride. After the homogenization, further preparation and measurement of lung tissue samples was performed identically to plasma samples. The analysis of the study samples was performed in compliance with the FDA guideline on “Bioanalytical Method Validation.”

### Influenza Virus Titration (AVICEL^®^ Plaque Assay)

MDCK.2 (ATCC: CRL-2936) and A549 (ATCC: CCL-185) cells were grown with MEM-medium (Minimal Essential Medium, 9.6 g/L, Gibco BRL) supplemented with 10% calf serum FCS and antibiotics (100 U/mL penicillin and 0.1 mg/mL streptomycin). For infection cells were grown overnight in 96-well plates (8 × 10^4^ cells/well). Immediately before infection the cells were washed with PBS and subsequently incubated with the different influenza A viruses at a MOI of 0.01 - 0.001 for 30 min at 37°C. After the 30 min incubation period the inoculum was aspirated and cells were incubated with either MEM (see above) supplemented with 0.2% BA instead of 10% FCS or MEM/BA medium containing different BAY 81-8781, ASA or SA concentrations. Supernatants were collected 24 h past infection.

To assess the number of infectious particles in the collected cell culture supernatants and mice lung homogenates, an AVICEL^®^ plaque assay was performed in 96-well plate format as described previously (Matrosovich et al., 2006). Virus-infected cells were immunostained by a 1 h incubation with a monoclonal antibody specific for the IAV nucleoprotein (AbD Serotec) followed by 30 min incubation with peroxidase-labeled anti-mouse antibody (DIANOVA) and 10 min incubation with True Blue^TM^ peroxidase substrate (KPL). After stopping the reaction with tap water the plates were dried and scanned with a resolution of 1200 dpi using the CANONFCAN 8800F scanner (Canon). To define the virus titer of the supernatants the plaques/foci of infected cells for every sample in each lane of the 96-well plates were counted. The virus titer is given as the logarithm to the base 10 of the plaques/foci mean value (pfu). The detection limit for this test was <1.7 log_10_ pfu/mL.

### Infection of Mice

For infection, the animals were anesthetized by intraperitoneal injection of 200 μl ketamine/rompun. Equal amounts of a 2% rompun (Bayer) and a 10% ketamine (Sanofi) stock solution were mixed at a rate of 1:10 with PBS. Mice were infected intranasally with adequate virus doses diluted in 50 μl BSS (buffered salt solution) by inoculating 25 μl into each nostril 1 h after treatment. The Institutional Animal Care and Use Committee of Tuebingen approved all animal studies. After infection mice were monitored twice daily and disease symptoms were scored: 0 = no symptoms; 1 = mild symptoms; 2 = medium symptoms; 3 = severe symptoms; 4 = death. In addition, bodyweight was measured once daily.

### Virus Titer Determination from Lungs of Infected Mice

Mice were sacrificed 24 h post infection and lungs were weighed, transferred into a Lysing Matrix D tube (MP Bio) and BSS was applied in an amount of the 10-fold volume of the lung. Organs were shredded using the FastPrep FP 120 (Savant). To remove the cell debris the homogenates were centrifuged for 15 min at 2000 rpm and the supernatant collected. The determination of virus titer in homogenates was performed using the AVICEL^®^ plaque assay described above.

### EC_50_ Determination

For the determination of the EC_50_, viral titers of the cell culture supernatants or mice lungs were calculated in percent. The number of pfu of the untreated virus-infected control was set as 100% and the titers of BAY 81-8781 treated samples were calculated as follows: Percent inhibition = 100/(pfu virus-infected sample × BAY 81-8781 treated sample).

The EC_50_ value (i.e., the concentration of BAY 81-8781 required to reduce the virus titer to 50%) was determined with the GraphPad Prism 5 Software by plotting the percent virus titer as a function of BAY 81-8781 concentration.

### Western Blot Analysis

For Western Blot analysis cells were lysed on ice with RIPA lysis buffer [1% (v/v) NP-40, 0.5% (v/v) DOC, 1% (w/v) SDS, 150 mM NaCl, 50 mM Tris pH 8, 90% H2O dest., 200 mM pefablock, 5 mg/mL aprotinin, 5 mg/mL leupeptin, 1 mM sodium-vanadate, 5 mM benzamidine] for 30 min. Cell lysates were cleared by centrifugation and protein yields were estimated employing a protein dye reagent (Bio-Rad Laboratories). Equal amounts of protein were separated by SDS-polyacrylamide gel electrophoresis and subsequently blotted on nitrocellulose membranes. Anti-PARP monoclonal antiserum was purchased from BD Transduction Laboratories. Antisera against the influenza virus proteins NS1 and PB1 were purchased from Santa Cruz Biotechnologies and monoclonal antibodies against NP and M1 were obtained from ABSerotec. Loading controls were performed with ERK2 antiserum (Santa Cruz Biotechnologies). Protein bands were visualized in a standard enhanced chemiluminescence reaction.

### RNA Isolation, Reverse Transcription, and Quantitative Real-time PCR

RNA from cells was isolated using the RNeasy Plus Mini Kit (Qiagen) according to manufacturers’ instructions. To synthesize cDNA 1 μg of total RNA were reverse transcribed using 0.5 μg oligo dT primer and 200 U Reversed Aid^TM^ H Minus Reverse Transcriptase (Thermo Fisher Scientific). For quantification of cDNA real-time PCR was performed using Brilliant III SYBR Green QPCR Master Mix (Agilent Technologies) and the Mx Pro 3005P cycler (Agilent Technologies). Changes in gene transcription were ascertained as differences between the transcription of the housekeeping gene GAPDH and the gene TRAIL using the 2^-ΔΔCT^ method (Livak and Schmittgen, 2001).

### Immunofluorescence Microscopy

A549 cells were grown on 15 mm glass plates. When 50% confluence was reached, cells were infected with IAV H5N1 strain A/Thailand/KAN-1/2004 (MOI = 5). Thirty minutes p.i., the inoculum was aspirated and medium/BA supplemented with BAY 81-8781 was added. Eight hours p.i., cells were washed twice with PBS, then fixed for 30 min with 3.7% paraformaldehyde (in PBS) at room temperature. After washing, cells were permeabilized with acetone, washed with PBS and blocked with 10% FBS in PBS for 20 min at 37°C. After blocking, cells were incubated with a monoclonal antibody against the viral NP (1:200) in PBS for 30 min. After further washes, cells were incubated with Alexa Fluor^®^ 594 chicken anti-mouse IgG; (1:300) in PBS for 30 min. Finally, cells were washed and mounted with Vectashield mounting medium with DAPI. Fluorescence was visualized using a Zeiss Axiovert 135 fluorescence microscope.

### Statistical Analysis

For investigation of the significance of the data, one way statistical analysis (ANOVA) followed by Bonferroni’s *post hoc* comparison test was performed using the GraphPad Prism 5 Software. Statistical analysis of the survival experiment was done with the Wilcoxon Signed Rank Test using the GraphPad Prism 5 Software. Statistical analysis of the virus reduction was done with the paired *t*-test using the GraphPad Prism 5 Software. Error bars represent standard errors of the mean (SEM). Statistics with a value of *p* < 0.05 were considered significant, *p*-values of < 0.05 are referred to as ^∗^, < 0.01 as ^∗∗^.

## Results

### BAY 81-8781 Exhibits Strong Anti-influenza Activity *in Vitro*

**FIGURE 1:**
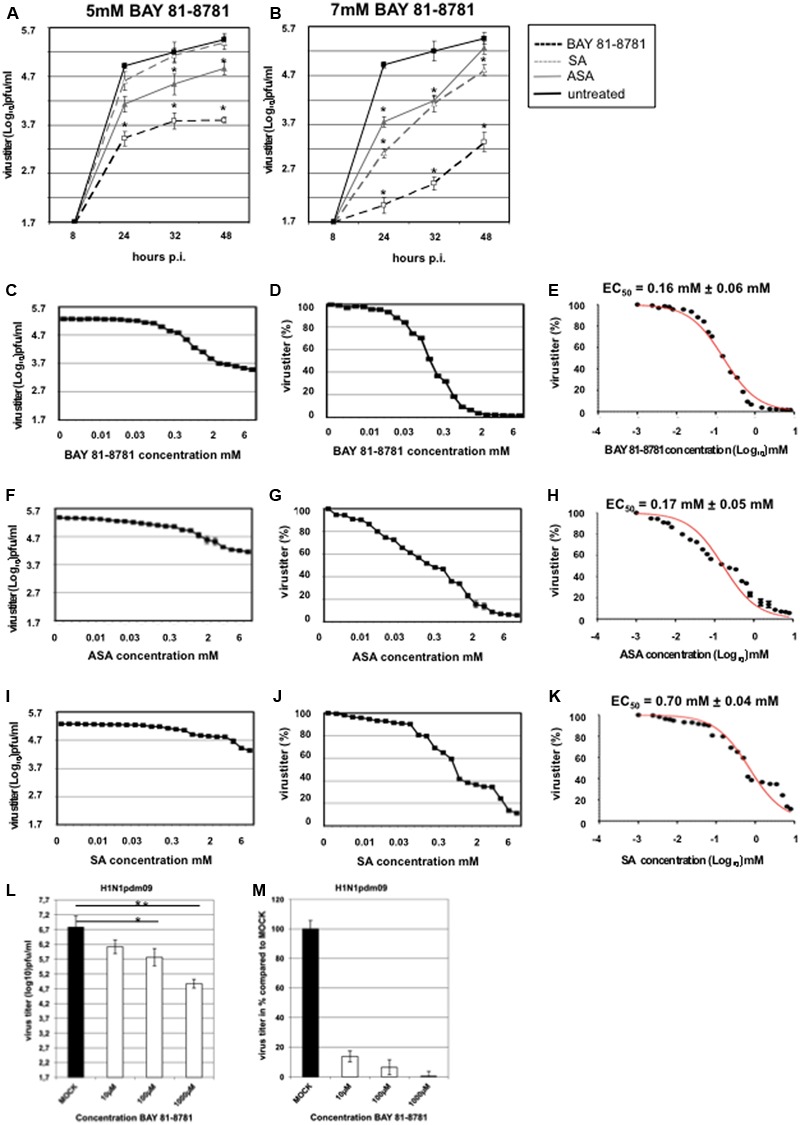
Antiviral activity of BAY 81-8781, ASA, and SA against H5N1 strain A/Mallard/Bavaria/1/2006 at different time-points after infection and EC_50_ value. **(A)** Treatment with 5 mM; BAY 81-8781 (open squares) or ASA (gray triangle) or SA (open triangle) and untreated MOCK-control (black squares). **(B)** Treatment with 7 mM; BAY 81-8781 (open squares) or ASA (gray triangle) or SA (open triangle) and untreated MOCK-control (black squares). Virus titers as triplicates are given in log_10_ pfu/mL. MOI = 0.01. For better comparison both treatment kinetics were performed in a single experiment. Therefore MOCK-control is the same in graph **A** and **B**. **(C–K)** A549 cells were used for infection with A/Mallard/Bavaria/1/2006 (MB1) (MOI: 0.001). At 30 min after infection cells were treated with 23 different concentrations of BAY 81-8781 **(C–E)**, ASA **(F–H)** or SA **(I–K)** ranging from 0.0024 mM to 8 mM. Twenty-four hours after infection supernatant was harvested and virus titer was determined as described in “Materials and Methods” section. **(C)** Virus titer was given in log_10_ pfu/mL and **(D)** in percent of untreated cells. **(E)** EC_50_ value was determined for BAY 81-8781 against MB1 infected A549 cells. **(F–H)** Virus titer in log_10_ pfu/mL, percent and EC_50_ value for ASA treated MB1 infected A549 cells and **(I–K)** virus titer in log_10_ pfu/mL, percent and EC_50_ value for SA treated MB1 infected A549 cells. Data represent one experiment, which was performed three times with similar results. **(L,M)** A549 cells were used for infection with A/Regensburg/D6/2009 H1N1pdm09 strain using a MOI of 0.001. Virus infected cells were either left untreated (MOCK) or were treated with 10, 100, or 1,000 μM BAY 81-8781. Twenty-four hours later the supernatant was harvested and progeny virus was determined as described in the “Materials and Methods” section. *p*-values of < 0.05 are referred to as ^∗^, < 0.01 as ^∗∗^.

The antiviral activity of BAY 81-8781, ASA, and SA against IAV was analyzed *in vitro* in cultured cells. Since a truly effective antiviral compound also must be active against aggressive and fast replicating viruses, the potency of the compound against a highly pathogenic and rapidly replicating H5N1 virus was chosen first. A549 cells were infected with influenza virus H5N1 strain A/Mallard/Bavaria/1/2006 and incubated with either 5 mM (**Figure 1A**) or 7 mM (**Figure 1B**) BAY 81-8781 (open squares), ASA (gray triangle) or SA (open triangle) and untreated MOCK-control (black squares). At different time-points as indicated supernatants were taken and virus titers were determined. Treatment with 5 mM BAY 81-8781 resulted in a strong and sustained reduction of virus titers at all time-points. An antiviral effect was also observed with ASA, but was less potent compared to BAY 81-8781. No antiviral effect was found upon SA treatment (**Figure 1A**, open triangle). Consistent with a dose dependent action 7 mM BAY 81-8781 resulted in an even stronger reduction of virus titers at all time-points. An antiviral effect was also found with ASA (gray triangles) and SA (open triangle) 24 h and 32 h post-infection which, however, was leveled-out at the 48 h time-point (**Figure 1B**).

To determine the EC_50_ values, different concentrations of the test compounds ranging from 0.0024 mM to 8 mM were used. The titers of infectious virus particles in the supernatant were determined by AVICEL^®^ plaque assay as described in “Materials and Methods.” All compounds reduced virus titer dose-dependently (**Figures 1C–K**). Virus titers are shown in log_10_ pfu [**Figure 1C** (BAY 81-8781), **Figure 1F** (ASA), **Figure 1I** (SA)] and in percent of untreated control [**Figure 1D** (BAY 81-8781), **Figure 1G** (ASA), **Figure 1J** (SA)]. The mean EC_50_ values 24 h after infection were 0.16 ± 0.06 mM for BAY 81-8781 (**Figure 1E**), 0.17 ± 0.05 mM for ASA (**Figure 1H**) and 0.7 ± 0.04 mM for SA (**Figure 1K**). Antiviral activity was confirmed against several field isolates of highly pathogenic avian H5N1 strains and H7N7 strains, but also against the human pandemic H1N1pdm09 strain. Here, even 10 μM of BAY 81-8781 was effective to reduce virus propagation (**Figures 1L,M**).

**FIGURE 2:**
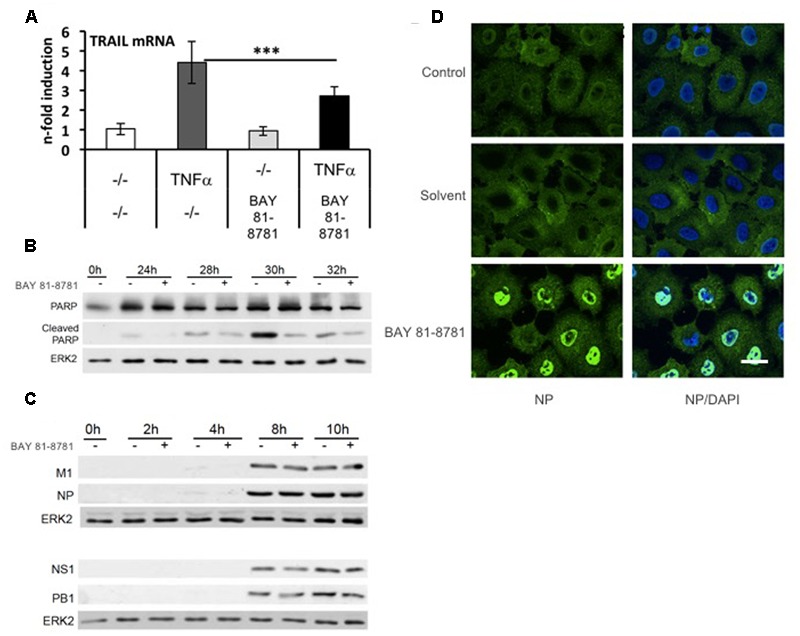
BAY 81-8781 has no effect on viral protein accumulation but leads to inhibition of caspase-mediated release of vRNPs from the nucleus. **(A)** BAY 81-8781 inhibits TRAIL mRNA expression. A549 cells were pretreated with 5 mM LASAG before addition of 20 ng/mL TNFα. Six hours post addition total RNA was isolated and subjected to real-time PCR to analyze TRAIL mRNA expression as *n*-fold of untreated control. Mean of three independent experiments is shown. **(B)** BAY 81-8781 inhibits virus induced caspase activity. A549 lung epithelial cells were left uninfected (lane 1) or infected with IAV A/FPV/Bratislava/79 (H7N7) (MOI = 0.01) for 24, 28, 30, and 32 h (lanes 2–9). Cells were then incubated in presence or absence of 5 mM BAY 81-8781. Cells were lysed at the respective time points p.i., protein lysates were separated by PAGE and blotted onto nitrocellulose membranes. Membranes were then incubated with antibodies against the major caspase 3 substrate poly ADP-ribose-polymerase (PARP). Increase of cleaved PARP correlates to caspase activation. An ERK2 blot served as a loading control. **(C)** BAY 81-8781 treatment does not affect accumulation of viral proteins within the first replication cycle. A549 lung epithelial cells were left uninfected (lanes 1) or were infected with A/FPV/Bratislava/79 (H7N7) (MOI = 5) for 2, 4, 8, and 10 h (lanes 2–9). Cells were then incubated in presence or absence of 5 mM BAY 81-8781. Cells were lysed at the respective time points p.i., protein lysates were separated by SDS-PAGE and blotted onto nitrocellulose membranes. Membranes were then incubated with antibodies against a representative set of viral proteins, including M1 (matrix protein), NP (nucleoprotein), NS1 (non-structural protein 1), and PB1 (polymerase basic subunit 1). ERK2 blots served as a loading control. **(D)** BAY 81-8781 treatment leads to a retention of vRNP complexes in the nucleus at late stages of the first replication cycle. A549 lung epithelial cells were infected with the human H5N1 Isolate A/Thailand/KAN-1/2004 (MOI = 5) for 8 h in presence or absence of 5 mM BAY 81-8781 or solvent. Cells were then fixed and stained with an antibody against the viral NP, the major constituent of vRNP complexes. After further incubation with Alexa Fluor^®^ 594-coupled secondary antibodies cells were subjected to immunofluorescence microscopy. In the right panel cells were additionally stained with the DNA intercalating agent DAPI to stain nuclei (in blue). Scale bar: 10 μm. *p*-value of < 0.005 is referred to as ^∗∗∗^.

**BAY 81-8781 has no effect on viral protein accumulation but leads to inhibition of caspase-mediated release of vRNP complexes from the nucleus.** In previous publications, it has been shown that in virus-infected cells inhibition of the NF-κB pathway result in impaired expression of proapoptotic factors such as FasL and TRAIL leading to an inhibition of virus-induced caspase activation (Wurzer et al., 2004; Ehrhardt et al., 2013). Since caspase activity in turn is required for efficient export of vRNPs from the nucleus (Wurzer et al., 2003), NF-κB inhibition leads to a nuclear retention of vRNPs (Wurzer et al., 2004; Mazur et al., 2007; Ehrhardt et al., 2013), which is the major molecular mode of action that determines antiviral activity of NF-κB inhibition. Here, we show that this is also the case for BAY 81-8781. TNFα induced activation of TRAIL mRNA is significantly reduced when cells were treated with 5 mM BAY 81-8781 (**Figure 2A**). BAY 81-8781 treatment of IAV infected A549 cells reduces the cleavage of the major caspase substrate PARP, indicative of a strong inhibition of caspase activation (**Figure 2B**). The compound does not impact accumulation of viral proteins indicating that early steps of the virus replication cycle are not affected (**Figure 2C**). In contrast, BAY 81-8781 had a strong effect on release of vRNPs from the nucleus (**Figure 2D**) as indicated by immunofluorescence studies of the viral NP, the major constituent of vRNPs. While in control and solvent treated cells vRNPs are predominantly found in the cytoplasm, the complexes are strongly retained and accumulated in the nucleus of BAY 81-8781 treated infected cells 6 h post infection. This fully confirms the earlier data generated with other NF-κB inhibiting agents.

### Analysis of the Treatment Window of Antiviral Action of BAY 81-8781

**FIGURE 3:**
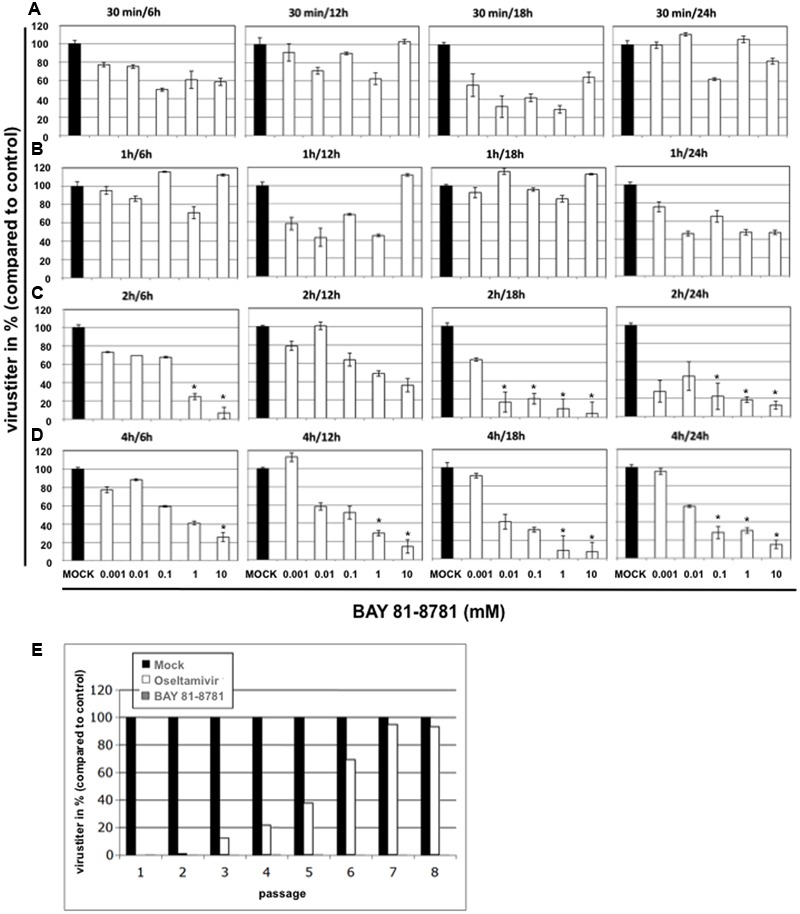
Time of addition kinetics with BAY 81-8781 and assessment of emergence of resistance. A549 cells were infected with A/Mallard/Bavaria/1/2006 (MB1) (MOI: 0.001). Twenty-four hours after infection cells were treated with five different concentrations of BAY 81-8781 ranging from 0.001 to 10 mM for either 30 min **(A)**, 1 h **(B)**, 2 h **(C)** or 4 h **(D)**. Either 6 h, 12 h, 18 h, or 24 h after infection supernatant was harvested. Virus titer was determined as indicated in the “Materials and Materials” section. Virus titer differed in controls depending on the time the supernatant was harvested after start of treatment. Therefore, only virus titer in percent compared to control is given in this figure, which allows the comparison of all graphs. **(E)** BAY 81-8781-treatment does not lead to emergence of resistant virus variant upon multiple passaging in cell culture. A549 lung epithelial cells were infected with IAV A/FPV/Bratislava/79 (H7N7) (FPV, fowl plague virus) at a MOI = 0.01 and incubated for 24 h with either BAY 81-8781 (5 mM) or oseltamivir (2 μM). Cell culture supernatants were removed and used to determine viral titers in plaque assays on MDCK cells. Supernatants were adjusted in titers and used for a second infection round in presence or absence of the inhibitors. This procedure was repeated until passage eight. Virus titers are shown relative to titers of untreated control cells (black bars) that were normalized to 100%. *p*-values of < 0.05 are referred to ^∗^.

To scrutinize the efficacy of antiviral action in more detail, time of addition experiments were performed. A549 cells were infected with H5N1 influenza virus A/Mallard/Bavaria/1/2006. Twenty-four hours post infection A549 cells were treated with different concentrations of BAY 81-8781 for different time periods, 30 min, 1 h, 2 h, or 4 h (**Figures 3A–D**). Thereafter, BAY 81-8781 was washed out and virus-containing supernatants were harvested 6 h, 12 h, 18 h, or 24 h after initial BAY 81-8781 treatment. When BAY 81-8781 was present for either 30 min or 1 h on H5N1 infected A549 cells, no antiviral effects were found even 24 h after onset of treatment (**Figures 3A,B**). In contrast, when BAY 81-8781 was present for 2 h or 4 h a dose dependent antiviral effect was found even 6 h after onset of treatment (**Figures 3C,D**). Nevertheless, this antiviral effect was more pronounced 18 h and 24 h after onset of treatment, where >80% virus titer reduction was found when using concentrations of either 1 mM or 10 mM (**Figures 3C,D**; 2 h/18 h, 2 h/24 h, 4 h/18 h, and 4 h/24 h). These results indicate that treatment of IAV infected cells with BAY 81-8781 for 2 h is sufficient for a strong antiviral effect.

### BAY 81-8781 Shows no Tendency to Induce Viral Resistance

A major advantage of a cell-directed antiviral compound versus a virus-directed drug would be that the virus cannot replace the missing cellular function. Thus, emergence of resistant variants should not occur. We therefore investigated, whether BAY 81-8781 treatment would result in the development of resistant IAV upon serial passaging. A549 lung epithelial cells were infected with IAV and incubated for 24 h with either 5mM BAY 81-8781 (**Figure 3E**, gray bars) or 2 μM Oseltamivir (**Figure 3E**, white bars). Cell culture supernatants were removed and viral titers were determined. Supernatants were used for further infection rounds in presence or absence of the inhibitors. This procedure was repeated until passage eight. All virus titers were normalized (100%) to untreated control cells (**Figure 3E**, black bars). Titers of oseltamivir treated virus-populations increased already after three passages, indicative of rising numbers of resistant virus variants. This number increased until passage 7, where resistance to oseltamivir was almost 100%. In contrast IAV showed no tendency of resistance to BAY 81-8781 in all eight passages (**Figure 3E**).

### Short Terminal Half-Lives of ASA and SA after Oral, i.v. or Inhaled Treatment in Mice

Pharmacokinetics of ASA, that was administered as BAY 81-8781, and SA were determined in plasma and lung homogenates of mice. After single inhalation, exposure to ASA was low in plasma and lung compared to SA. Plasma concentrations of both ASA (*C*_max_: 2.19 mg/L) and SA (*C*_max_: 61.7 mg/L) were higher than in lung homogenate (ASA, *C*_max_: 0.58 mg/L; SA, *C*_max_: 16.8 mg/L). Bioavailability of ASA as measured by calculating the area under curve (AUC) of the drug concentration over time was low for plasma (AUC: 1.35 mg^.^h/L) and lung (AUC: 0.329 mg^.^h/L) (**Table 1**). The relative bioavailability (*F*) compared to i.v. treatment was 14.0%. After oral administration of 15 mg/kg BAY 81-8781, bioavailability was low and comparable to the bioavailability observed after inhalation probably due to a high first-pass effect. Plasma (*C*_max_: 1.29 mg/L) and lung (*C*_max_: 0.251 mg/L) concentrations of ASA were low compared to SA (Plasma *C*_max_: 51.9 mg/L; Plasma *C*_max_: 12.5 mg/L). Plasma exposure was higher than in lung homogenate both for ASA and SA (**Table 1**). After intravenous administration plasma clearance was high for ASA, and volume of distribution at steady state was moderate. Terminal half-lives were short for ASA and SA. In plasma, AUC and *C*_max_ were about 25-fold and 2.5-fold higher for SA compared to ASA, respectively. Measured ASA concentrations in lung homogenate were very low, suggesting that ASA degradation to SA was considerable before stabilization of the samples (**Table 1**). Furthermore, a similar AUC for lung homogenate after i.v and oral administration is inconsistent considering a bioavailability of about 10%.

**Table 1:** Pharmacokinetics of ASA and SA.

Matrix		Plasma		Lung	
Compound		ASA	SA	ASA	SA
**Inhalation**					
Dose	[mg/kg]	59	45.2	59	45.2
AUC	[mg⋅h/L]	1.35	138	0.329	38.2
AUC_norm_	[kg⋅h/L]	0.023	3.05	0.006	0.84
*C*_max_	[mg/L]	2.19	61.7	0.58	16.8
*C*_maxnorm_	[kg/L]	0.04	1.36	0.01	0.37
*t*_1/2_	[h]	0.46	0.89	0.21	1.0
Interval^a^	[h]	0.9–2.4	1.4–4.4	0.57–1.4	1.4–4.4
*F*^b^	[%]	14.0	nc	nc	nc

**Oral**					
Dose	[mg/kg]	15	11.5	15	11.5
AUC	[mg⋅h/L]	0.253	58.6	0.0688	15.3
AUC_norm_	[kg⋅h/L]	0.0169	5.09	0.00439	1.33
*C*_max_	[mg/L]	1.29	51.9	0.251	12.5
*C*_maxnorm_	[kg/L]	0.0861	4.51	0.0167	1.09
*t*_max_	[h]	0.0833	1.00	0.250	0.250
*t*_1/2_	[h]	0.144	0.596	0.0757	0.498
Interval^a^	[h]	0.25–1.0	2.0–5.0	0.25–0.5	0.5–2.0
*F*^b^	[%]	10.3	nc	nc	nc

**i.v.**					
Dose	[mg/kg]	20	15.3	20	15.3
AUC	[mg⋅h/L]	3.27	80.6	0.0781	nc
AUC_norm_	[kg⋅h/L]	0.163	5.28	0.00391	nc
*C*_max_	[mg/L]	26.8	69.8	0.297	21.4
*C*_maxnorm_	[kg/L]	1.34	4.56	0.0148	1.40
*t*_1/2_	[h]	0.112	0.525	0.2970	nc
Interval^a^	[h]	0.083–1.0	0.5–2.0	0.083–1	nc

*^*a*^Used for regression to determine half-live. ^*b*^Related to the AUC_*norm*_ 20 mg/kg i.v.*

### BAY 81-8781 Only Acts Antiviral When Administered via Inhalation But Not via the Oral Route

**FIGURE 4:**
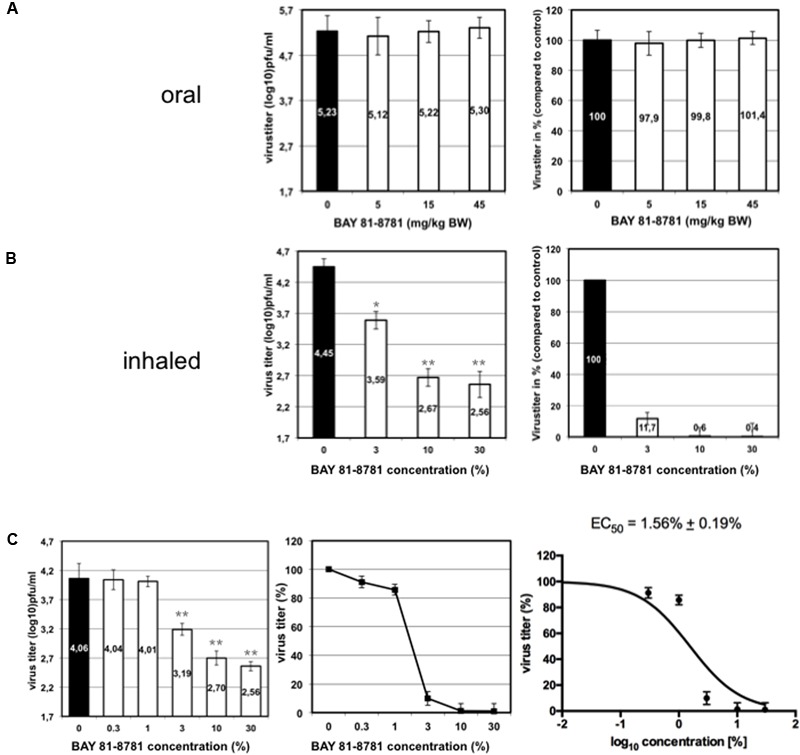
Virus titer in the lung of mice treated either orally or by inhalation. Eight weeks old C57BL/6 mice (four per group) were anesthetized with ketamine/rompun and infected with 1.5 × 10^5^ PFU (5x MLD_50_) of the influenza virus strain A/FPV/Bratislava/79 (H7N7). Starting 1 h prior to infection mice received a single BAY 81-8781 treatment either *per os* **(A)** or via inhalation **(B)**. Oral treatment was performed with, either 5, 15, and 45 mg/kg bodyweight, while 35 mL ddH_2_O containing either 3%, 10%, or 30% of BAY 81-8781 were used for aerosol treatment. Treatment was performed as described in “Materials and Methods.” Mice were sacrificed 24 h post infection. The determination of virus titer in lung-homogenates was performed using the AVICEL^®^ plaque assay. Results are presented as virus titer (log_10_) pfu/mL (left) or % virus titer (right). The experiment was performed three times independently. **(C)** Dose response curve of virus titer in the lung of mice treated with inhaled BAY 81-8781. Eight weeks old C57BL/6 mice (four per group) were anesthetized with ketamine/rompun and infected with 1.5 × 10^5^ PFU (5x MLD_50_) of the influenza virus strain A/FPV/Bratislava/79 (H7N7). Starting 1 h prior to infection mice received 35 mL ddH_2_O treatment via inhalation of either 0% (solvent control), 0.3%, 1%, 3%, 10% and 30% BAY 81-8781. Treatment was performed as described in “Materials and Methods.” Mice were sacrificed 24 h post infection. The determination of virus titer in lung-homogenates was performed using the AVICEL^®^ plaque assay. Results are presented as virus titer (log_10_) pfu/mL (left) or % virus titer (middle). EC_50_ was calculated using graph prism software (right). *p*-values of < 0.05 are referred to ^∗^ and *p*-values of < 0.01 are referred to ^∗∗^.

We compared the antiviral effect of BAY 81-8781 in IAV infected mice after inhalation and oral administration. C57BL/6 mice were infected with 1.5 × 10^5^ PFU (5x MLD_50_) of a mouse adapted highly pathogenic influenza virus strain. Starting 1 h prior to infection mice received a single BAY 81-8781 dose either *per os* (5, 15, and 45 mg/kg bodyweight; **Figure 4A**) or via inhalation (3, 10, and 30%; **Figure 4B**). Inhalation was performed as described in “Materials and Methods.” Mice were sacrificed 24 h post infection and virus titers were determined from mouse lungs. As shown in **Figure 4A** no reduction of virus titers was found when mice were treated via the oral route (**Figure 4A** left: virus titers in log_10_ pfu/mL, right virus titers in %). In contrast, when mice were treated by inhalation, already a 3% BAY 81-8781 solution was sufficient to reduce virus titers by almost one log_10_ pfu/mL (**Figure 4B**). Titer reduction could be further increased up to almost two log_10_ pfu/mL when higher BAY 81-8781 concentrations of 10% or 30% were used (**Figure 4B**). These results indicate that inhalation is an effective route of BAY 81-8781 administration.

Next, we determined the EC_50_ value in C57BL/6 mice against the prototype mouse adapted H7N7 IAV strain. Infected mice were treated with different concentrations of BAY 81-8781 as indicated in **Figure 4C**. One hour prior to infection mice received BAY 81-8781 treatment. This treatment was repeated 12 h after infection. Twenty-four hours after infection the animals were sacrificed, lungs were taken and virus titers were measured. As shown in **Figure 4C**, the antiviral effect of BAY 81-8781 against H7N7 IAV was dose dependent with an EC_50_ value of 1.56% ± 0.19% BAY 81-8781. This result is in line with the experiment presented in **Figure 4B**, since an efficient titer reduction was already observed, when a BAY 81-8781 concentration of 3% was used.

### BAY 81-8781 Treatment Protects Mice from Lethal Virus Challenge

**FIGURE 5:**
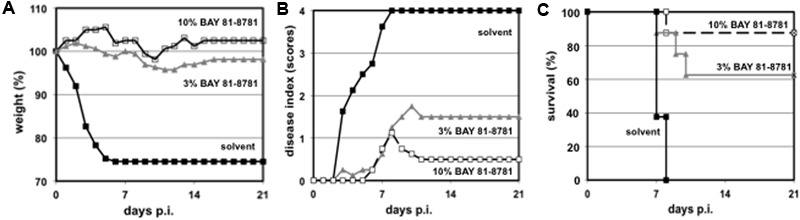
**(A)** Bodyweight, **(B)** disease symptoms, and **(C)** survival of H5N1 infected mice treated with 3% and 10% BAY 81-8781. Eight weeks old C57BL/6 mice (four per group) were anesthetized with ketamine/rompun and infected with 1.5 × 10^5^ PFU (5x MLD_50_) of the influenza virus strain (H5N1; MB1). Starting 1 h prior to infection mice received treatment via inhalation of 35 mL ddH_2_O either 0% (solvent control, black squares), 3% (gray triangles), or 10% (open squares) of BAY 81-8781 twice daily. Mice were observed twice daily. Bodyweight prior to the first treatment was set as 100%. Disease was scored as described in Section “Materials and Methods.” When mice died or needed to be killed because of severe disease or increased weight loss (according to the German animal protection law) last measured bodyweight was carried throughout the end of the observation period.

The results above demonstrate a strong antiviral effect of BAY 81-8781 on progeny virus when administered to the lungs of infected mice. Now the question arises, whether this reduction of virus titers in the lung would also influence the clinical outcome of the disease and would prevent death in otherwise lethal infection with a highly pathogenic influenza virus strain. C57BL/6 mice were infected with H5N1 virus and treated BID, starting 1 h prior to infection and thereafter every 12 h for a total of 5 days. Control animals were treated with solvent alone. These animals immediately started in losing weight 1 day after infection. This weight loss continued until the animals died or had to be killed due to animal care regulation rules (**Figure 5A**, black squares). In contrast only a moderate weight loss was found when mice were treated with 3% BAY 81-8781 (**Figure 5A**, gray triangle) or 10% BAY 81-8781 (**Figure 5A**, open square). A similar picture was found for disease progression. While solvent treated animals rapidly developed severe disease (**Figure 5B**, black squares), the disease was rather moderate in BAY 81-8781 treated animals but increased in mice treated with 3% BAY 81-8781 (**Figure 5B**, gray triangle) compared to animals receiving 10% BAY 81-8781 (**Figure 5B**, open square). 10% BAY 81-8781 treatment protected 7/8 mice (87.5%) from death, while 5/8 mice (62.5%) were protected by 3% BAY 81-8781 treatment. In contrast and as expected all mice treated with solvent alone died between days 6–7 p.i. (**Figure 5C**).

BAY 81-8781 treatment of influenza virus infected C57BL/6 mice was effective, when started 1 h prior to infection, demonstrating the prophylactic potential of BAY 81-8781. Based on the *in vitro* experiments (**Figures 3A–D**) one might speculate about a therapeutic potential. Therefore, mice were infected and treatment started as late as 48 h after infection. Moreover, the efficiency was compared to Tamiflu^®^ treatment starting also 48 h after infection. It is well known that Tamiflu^®^ is very effective in protecting mice when administered shortly after infection. The efficiency when given at later time-points after infection is weak. Since Tamiflu^®^ was administered orally two different control groups were used (**Table 2**). Both drugs were administered BID every 12 h for 5 days. The observation period was 21 days. While solvent treated animals developed disease symptoms and lost weight very fast mice treated with 10% BAY 81-8781 either showed no or only slight weight loss and marginal disease symptoms. Four of eight mice (50%) survived the infection. The BAY 81-8781 treated mice that were not protected from lethal infection died at an average of 8.8 days p.i., 2 days later compared to the solvent animals (**Table 2**). Tamiflu^®^ treated animals were not protected from lethal disease and all mice developed disease, lost weight and died comparable to the solvent treated mice. Thus, BAY 81-8781 treatment showed a greater therapeutic window as Tamiflu^®^ treatment against infections with a highly pathogenic influenza virus.

**Table 2:** Survival after treatment of Aspirin^®^ inhale or Tamiflu^®^ starting 48 hpi.

	Survival (survival/total)	Day of death (p.i.)
Solvent (p.o.)	0/8	7.4 ± 1.2
Solvent (inhalation)	0/8	6.8 ± 0.7
Tamiflu^a^	0/8	7.1 ± 1.4
BAY 81-8781^b^	4/8	8.8 ± 1.3

*^*a*^Tamiflu was given BID via the oral route of administration. ^*b*^10% BAY 81-8781 was given BID via inhalation.*

## Discussion

Influenza virus replication is dependent on the activation of the NF-κB signaling pathway (Nimmerjahn et al., 2004; Ludwig and Planz, 2008; Planz, 2013; Keener, 2017). We and others described that ASA inhibits influenza virus replication in cell culture and in a mouse model (Huang and Dietsch, 1988; Mazur et al., 2007). This is because ASA inhibits the NF-κB signaling pathway by specifically interfering with the IκB kinase beta (Yin et al., 1998). *In vivo* antiviral effects were found, when ASA was delivered directly into the lung using a nebulizer system (Mazur et al., 2007). Nevertheless, the formulation used in this experimental inhalation therapy is not suitable for clinical usage, since ASA is degraded rapidly and would cause respiratory irritations due to its acidic nature. D,L-lysine acetylsalicylate ⋅ glycine (BAY 81-8781, Aspirin^®^ inhale) is more stable and highly soluble in water. It dissociates readily into ASA and the amino acids lysine and glycine upon dissolution in aqueous media. Thus, BAY 81-8781 could have an advantage compared to ASA for treatment against IAV via inhalation. In the present work, we tested the ability of BAY 81-8781 to function as an antiviral against IAV and we scrutinized the mode of action in more detail.

Due to the rapid dissolution of lysine and glycine of BAY 81-8781 it was expected that BAY 81-8781 would have similar functions in inhibiting NF-κB as ASA. This similar mode of action was confirmed by reduced TRAIL expression (**Figure 2A**), which is regulated by the NF-κB transcription factor and enhances IAV propagation in an autocrine and paracrine fashion (**Figures 2A,B**). This similar mode of action was also confirmed by *in vitro* pharmacodynamic studies, where equal EC_50_ values were determined for BAY 81-8781 (**Figure 1E**) and ASA (**Figure 1H**) for H5N1 influenza virus. In contrast, the EC_50_ value for SA in the same assay was 4.2 times less (**Figure 1K**; EC_50_: 0.7 mM). Interestingly, when antiviral activity over time of BAY 81-8781, ASA, and SA were compared 5 mM BAY 81-8781 treatment was superior to ASA or SA. Using 7 mM further increased this antiviral effect (**Figures 1A,B**). This clearly increased antiviral activity of BAY 81-8781 over ASA might be due to advanced stability properties even though lysine and glycine rapidly dissolute in aqueous media. BAY 81-8781 does not interact with the virus directly, since viral protein synthesis is not altered (**Figure 2C**). Caspase cleavage is required for export of vRNP complexes. This cleavage is inhibited by BAY 81-8781 treatment as monitored by caspase downstream target PARP (**Figure 2B**). Thus, in addition to ASA (Wurzer et al., 2004) also BAY 81-8781 treatment leads to retention of vRNP complexes in the nucleus during IAV replication cycle (**Figure 2D**).

Concerning the treatment window of its antiviral properties, we found that BAY 81-8781 can be applied to IAV infected cells for only 2 h and an antiviral activity was still found. This is an important result also from a pharmacokinetic point of view. Since H5N1 is a highly pathogenic and fast replicating IAV, this time-window might even be prolonged using other IAV strains. Nevertheless, we preferred to work with these highly pathogenic strains of the H7N7 and H5N1 origin to further strengthen the meaningfulness of the present work. Thus, taken the *in vitro* data together, although almost identical EC_50_ values were determined for BAY 81-8781 and ASA using this short time assays, the antiviral effect of BAY 81-8781 seems to be prolonged in cell culture system compared to ASA. In this line, IAV shows also no tendency to develop resistant viral mutants compared to oseltamivir treatment (**Figure 3E**). In general oseltamivir is very potent against different influenza virus strains, but passaging of virus in the presence of oseltamivir leads to a mutation in the viral neuraminidase and consequently to the development of oseltamivir resistant mutants.

To get a better understanding of the *in vivo* properties of BAY 81-8781, the pharmacokinetics of ASA (BAY 81-8781) and its metabolite SA were studied in mice (pharmacology species) after single oral, intravenous, and inhaled administrations. It is worth noting that in plasma and lung ASA is present even after BAY 81-8781 treatment due to the rapid dissolution of lysine and glycine. After inhalation, amounts of ASA in plasma and lung was low compared to SA. The high clearance of ASA is due to the rapid hydrolysis by esterases present in plasma, red blood cells, liver, and lung. The exposure in plasma was higher than in lung homogenate for both ASA and SA with AUC and *C*_max_ about four-fold higher in plasma. However, for technical reasons (much longer time for sample preparation compared to plasma), lung homogenate concentration values should in general be interpreted with caution. *C*_max_ of SA was about three-fold higher in plasma than in lung. Bioavailability was low for ASA (14%) and comparable to the bioavailability determined after oral administration. Dose-normalized AUC and *C*_max_ of ASA in lung homogenate were similar after inhalation and oral administration (**Table 1**). The pharmacokinetic studies in mice revealed that systemic exposure after inhalation and oral administration was generally similar. In addition, no marked differences were observed for the local exposure in lung homogenate, but statements concerning the local distribution in the lung, which might be different, cannot be made. However, drawing conclusions from the measured lung homogenate concentrations about actual concentrations is highly restricted due to cleavage of ASA during sample preparation. Since no objections have emerged from the pharmacokinetic studies to perform studies with ASA formulated as BAY 81-8781 (BAY 81-8781; Aspirin^®^ Inhale) administered by inhalation, we compared oral and inhaled treatment for antiviral activity. It was surprising for us that oral treatment had no antiviral effect. In contrast, an antiviral effect was found after inhalation of Aspirin^®^ Inhale using a nose only device (**Figures 4A,B**). As mentioned above we have no valuable data on ASA distribution in the lung (or upper respiratory tract) after oral and inhaled treatment due to technical limitations. Nevertheless, from the results shown in **Figure 4** one is tempted to speculate that indeed the ASA (BAY 81-8781) distribution in the lung is different after inhalation compared to oral treatment. Thus, inhalation is a prerequisite for successful antiviral effect *in vivo*. The *in vivo* EC_50_ value was achieved when mice inhaled a 1.5% BAY 81-8781 solution for 20 min. 0.5g LASAG BAY 81-8781 were dissolved in 35 mL ddH_2_O. The deposition is roughly 1% and only a total of roughly 5 mL of the solution reached the four mice in the nose only device. Thus, the actual BAY 81-8781 deposition in the lung of a single mouse needed to be 180–200 μg to achieve the EC_50_.

Also, a clear protection of mice was found after lethal IAV challenge (**Figure 5**) and this effect was also found, when treatment started 48 h after infection, a time point where Tamiflu^®^ was unable to protect mice from lethal IAV challenge. In contrast, at least 50% of the BAY 81-8781 treated mice survived and the non-survivors died 2 days later compared to controls (**Table 2**). During all animal experiments, even with high doses of 30% no adverse events were found.

Taken together, BAY 81-8781 represents a novel, very promising candidate for an anti-influenza drug with several advantages compared to the standard of care. The compound is very well tolerated and because of its safety profile and the wide clinical experience with the active core compound, BAY 81-8781 could not only serve as a therapeutic agent but even be considered for prophylactic use. The fact that BAY 81-8781 targets a cellular rather than a viral component would prevent the emergence of resistance. Finally, our data indicate a much wider treatment window compared to the standard of care, which is a crucial issue in influenza management. Since there is still an urgent need for new antivirals against influenza, BAY 81-8781 could perfectly meet this medical need and is likely to have a better systemic safety profile.

## Author Contributions

KD, EH, SD, CE, and OP performed experiments. GS, KN, SC, GvD, SL, and OP designed the experiments. SC, GvD, SL, and OP prepared the manuscript.

## Conflict of Interest Statement

GS reports personal fees from Activaero/Vectura/Ventaleon GmbH, during the conduct of the study and personal fees outside the submitted work. GS, SC, KN, OP, SP, SL are shareholders from Activaero/Vectura. KN reports personal fees from Ventaleon GmbH outside the submitted work. OP reports grants from Activaero/Vectura/Ventaleon GmbH, during the conduct of the study; also grants from Atriva Therapeutics GmbH and Activaero/Ventaleon GmbH outside the submitted work. SC, OP, SL, SP are shareholders from Atriva Therapeutics GmbH outside the submitted work. In addition, SL, SP, OP have a patent 8313751 and a patent EP20090 701974 both issued to Ventaleon GmbH. GS has an issued patent WO 2009/089822 A2. The other authors declare that the research was conducted in the absence of any commercial or financial relationships that could be construed as a potential conflict of interest.
